# Ultrasound Findings in Hand Joints Involvement in Patients with Psoriatic Arthritis and Its Correlation with Clinical DAS28 Score

**DOI:** 10.1155/2015/353657

**Published:** 2015-12-22

**Authors:** Priyanka Naranje, Mahesh Prakash, Aman Sharma, Sunil Dogra, Niranjan Khandelwal

**Affiliations:** ^1^Department of Radiodiagnosis, Postgraduate Institute of Medical Education and Research (PGIMER), Chandigarh 160012, India; ^2^Department of Internal Medicine, Postgraduate Institute of Medical Education and Research (PGIMER), Chandigarh 160012, India; ^3^Department of Dermatology, Venereology and Leprology, Postgraduate Institute of Medical Education and Research (PGIMER), Chandigarh 160012, India

## Abstract

*Objective*. To determine the frequency of the various ultrasound findings in hand joints in patients with psoriatic arthritis and correlate grayscale and Power Doppler ultrasonography findings with Disease Activity Score 28.* Methods*. This prospective study was performed in 30 patients. Ultrasound evaluation of 28 joints of both hands was undertaken and various findings were recorded including synovial hypertrophy, Power Doppler abnormality, soft tissue thickening, tendonitis, joint effusion, periosteal reaction, and erosions. Composite ultrasound scores and Disease Activity Score 28 were calculated and compared. Spearman correlation was used to see relationship between the ultrasound and DAS28 scores.* Results*. Ultrasound detected more abnormalities in the hand joints than did clinical examination. The frequency of various ultrasound abnormalities was as follows: Synovial hypertrophy was seen in 100%, Power Doppler abnormality suggesting hypervascularity was seen in 36.7%, soft tissue thickening was seen in 66.7%, periosteal reaction was seen in 33.3%, erosions were seen in 30% (mostly in DIP and PIP joints), and flexor tendonitis was seen in 6.7% of patients. Significant correlation was found between Disease Activity Score 28 and grayscale joint score (GSJS) (Spearman's *ρ*: 0.499; *P*: 0.005), grayscale joint count (GSJC) (*ρ*: 0.398; *P*: 0.029), and Power Doppler joint score (PDJS) (*ρ*: 0.367; *P*: 0.046). There was a statistically significant difference between remission and low disease activity group and moderate disease activity group in terms of GSJC, GSJS, PDJC, and PDJS (*P* < 0.05). These ultrasound measures were higher in moderate disease activity zone patients.* Conclusion*. Ultrasound is a useful modality for the objective assessment of psoriatic arthritis. Ultrasound including Power Doppler can be used as a modality for assessment of severity of psoriatic arthritis as it correlates with the clinical scoring.

## 1. Introduction

Psoriatic arthritis (PsA) is commonly described as chronic, seronegative, inflammatory spondyloarthropathy seen in association with psoriasis. It has been shown that psoriatic arthritis patients may develop progressive joint damage, deformity, and disability [1]. Progression of the psoriatic arthritis adds to increase in morbidity. PsA occurs in a variable, albeit considerable, percentage of psoriatic patients that range from 10 to 30% depending on the studied population [2]. In India, prevalence of PsA in patients with psoriasis has been around 7 to 8% [3]. The arthropathy generally begins several years after the commencement of the disease [4, 5]. Serological investigations for RA are negative in most of these patients [6].

Among various imaging modalities, ultrasound (US) is routinely accessible, inexpensive, and noninvasive diagnostic imaging modality. It is a rapidly evolving technique that is gaining an increasing success in the assessment of PsA. It provides measures of synovial morphology and vascularity. Grayscale (GS) ultrasound visualizes the structures of the joint and can discriminate amongst synovial hypertrophy and various other sources of perceptible swelling of the joint such as tenosynovitis or edema in subcutaneous plane [7]. Power Doppler (PD) sonography shows increased soft tissue vascularity with superior sensitivity and hence differentiates inflamed from noninflamed synovial swelling. Various scoring systems have been elicited to clinically assess the disease activity and severity in psoriatic arthritis, out of which Disease Activity Score 28 (DAS28) has shown to be of better accuracy and DAS28 is more suitable to assess PsA forms with joint involvement [8]. On an individual joint basis there has been a poor correlation between joint tenderness and swelling which are the clinical presentations of synovitis and grayscale as well as Power Doppler ultrasound measures of synovial pathology [9]. Previous studies done in patients with rheumatoid arthritis (RA) have revealed that US identifies more synovial changes than is clinically noticed and PD depicts that it is not essential for all the swollen joints to show hypervascularity and that yet a clinically normal joint may show synovial hypervascularity [9, 10]. It has been tried to overcome these differences between clinical and US findings in a particular joint in patients with RA by using composite counts or scores from many joints. Such scores would overall become indicative of entire disease activity in a patient [11]. The association between Power Doppler ultrasonography and clinical scores has not been evaluated in PsA. Therefore this study was done to assess the relation between DAS28 score and its components with compound GS and PD Ultrasound counts and scores of the metacarpophalangeal (MCP), proximal interphalangeal (PIP), and distal interphalangeal (DIP) joints in psoriatic arthritis.

## 2. Material and Methods

The Ethical Committee of Postgraduate Institute of Medical Education and Research (PGIMER), Chandigarh, approved this study. Written informed consent was taken from all controls and patients. The prospective study was carried out on the patients presenting to the rheumatology outpatient department (OPD) of PGIMER, Chandigarh. A total of 30 consecutive patients diagnosed to have psoriatic arthritis and presenting with symptoms of pain/swelling of the small joints of hands were included. The study was conducted in the Department of Radiodiagnosis and Imaging, in collaboration with Department of Internal Medicine, and Department of Dermatology, PGIMER, Chandigarh.

### 2.1. Inclusion Criteria

Patients diagnosed to have psoriatic arthritis according to ClASsification criteria for Psoriatic ARthritis (CASPAR) and presenting with pain and/or swelling of the small joints of hands were incorporated in the study.

### 2.2. Exclusion Criteria

Exclusion criteria included patients with symptoms of arthritis before the onset of skin disease and patients with rheumatoid factor positivity and coexistent infection of the digits and amputation/injury/plaster of any digit.

Detailed history was taken and complete clinical examination was done at the time of enrolment. For each participant, diagnosis of psoriatic arthritis was established by a rheumatologist in the rheumatology clinic by patient interview including history of pain and/or swelling of hand joints. Clinical examination was performed to assess for the swollen joints and tender joints. Erythrocyte sedimentation rate was determined by using Wintrobe's method on the same day of the clinical examination and Visual Analog Scale (VAS) was used for the patient's assessment of general health (GH).

DAS28 score was calculated to assess the disease activity of PsA.

### 2.3. Calculation of DAS28 Score

Examination of swollen and tender joints of patient was performed and each affected joint was noted. Sum of all swollen and tender joints was done and totals were recorded. Patient's erythrocyte sedimentation rate (ESR) was recorded in mm/h. Visual Analog Scale (VAS) of 100 mm was used to record the general health of the patient. DAS28 value was calculated using the following formula. A DAS28 calculator v1.1-beta was used to compute this value:

(1) DAS28=0.56∗√tender  joints+0.28∗√swollen  joints+0.70∗LnESRCRP+0.014∗VAS.

On the same day of clinical evaluation, ultrasound was performed in bilateral metacarpophalangeal, proximal interphalangeal, and distal interphalangeal joints using Philips HD 11 system or Philips iU22 system, equipped with a 3 to 12 MHZ linear transducer. For Power Doppler studies, “low flow” settings with a medium to low wall filter (to minimize flash artifact) were used and a pulse repetition frequency of 500 to 700 Hz was set. The color gain was accustomed to just below the noise level. Each joint was scanned in both transverse and longitudinal planes for grayscale and Power Doppler study.

**Figure 1:**
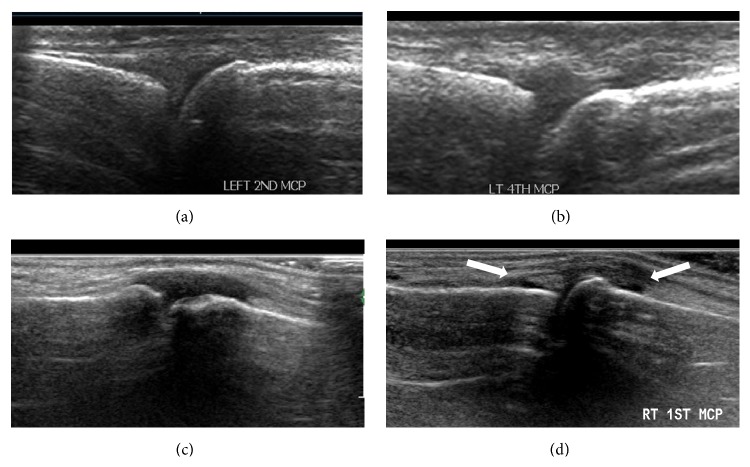
(a–d) Grading of synovial thickening is depicted; (a) Grade 0, (b) Grade 1, (c) Grade 2, and (d) Grade 3. Arrows indicate extension of synovial thickening to both metadiaphyses.

In every patient, scanning of 28 hand joints (10 MCP, 8 PIP, and 10 DIP joints) was done and the presence of abnormal synovial thickening, soft tissue thickening, tendonitis, periosteal reaction, joint effusion, erosions, and Power Doppler abnormality was noted. In addition, these joints were scored according to Table 1. Interphalangeal joint of first digit was considered as DIP joint. The severity grade of GS score was determined according to the following grading of synovial thickness (Figure 1) [12]:

 Grade 0: no/minimal synovial thickening (considered normal).
 Grade 1: synovial thickening bulging over the line joining the tops of the bones forming the joint without extension along the bone diaphyses.
 Grade 2: synovial thickening extending to one of the metadiaphyses.
 Grade 3: extension to both metadiaphyses.

Separate grayscale and Power Doppler subjective score were documented for each joint which ranged from 0 to 3. Scores of 1, 2, and 3 were considered abnormal, and 0 was considered as normal. These scores were used to derive the composite US measures of synovial pathology as follows: (i) grayscale joint count (GSJC): number of joints scoring either 1, 2, or 3, out of a total of 28; (ii) grayscale joint score (GSJS): sum of the GS scores in all 28 joints, out of a total of 84; (iii) Power Doppler joint count (PDJC): number of joints scoring either 1, 2, or 3, out of a total of 28; and (iv) Power Doppler joint score (PDJS): the sum of the PD scores in all 28 joints, out of a total of 84.

Accordingly, the GSJC and PDJC represented number of normal or abnormal joints, similar to TJC and SJC system employed in the DAS28, whereas the GSJS and PDJS suggested an assessment of severity. The correlation between US measures and clinical measures of the disease was determined amongst the following variables: the US measures and the DAS28 score and the DAS28 components, including SJC, TJC, ESR, and VAS.

The statistical analysis was performed using Statistical Package for Social Sciences (SPSS Inc., Chicago, IL, version 17.0 for Windows). Spearman correlation was used to see relationship between different continuous variables. Qualitative or categorical variables were described as frequencies and proportions (percentages). All statistical tests were two-sided and performed at a significance level of *α* = 0.05.

## 3. Results: Psoriatic Arthritis Patients

### 3.1. Demographic Data of the Patients (Table 2)

Thirty patients (16 males and 14 females) were enrolled in the study. The mean age was 38.97 ± 12.187 years (range 18–62 years). None of the patients had a family history of psoriasis. On examination, 8/30 patients (26.7%) had nail involvement due to psoriasis. 25/30 (83.3%) patients were on medical treatment in the form of methotrexate, steroids, or nonsteroidal anti-inflammatory drugs (NSAIDs).

### 3.2. Range of US Measures and Clinical Measures of Synovial Disease

Wide range of scores was seen in both clinical and US measures of synovial pathology (Table 3) in 30 recruited patients, indicating a wide spectrum of disease activity in them.

**Figure 2:**
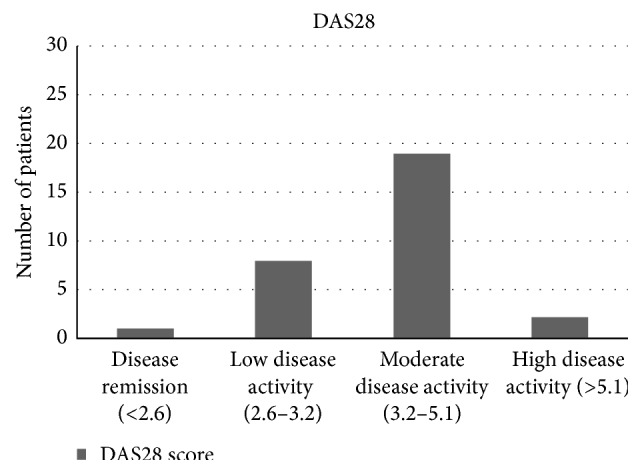
Distribution of patients in DAS28 subcategories.

Most of the patients (19) were having DAS28 score within the moderate disease activity range, nine (9) patients were in remission and low disease activity, and only two (2) patients were in high disease activity zone, as shown in Figure 2.

In the 30 patients, a total of 840 MCP, PIP, and DIP joints were evaluated clinically and sonologically. Out of this total, clinically 50 were swollen joints and 113 were tender joints.

**Figure 3:**
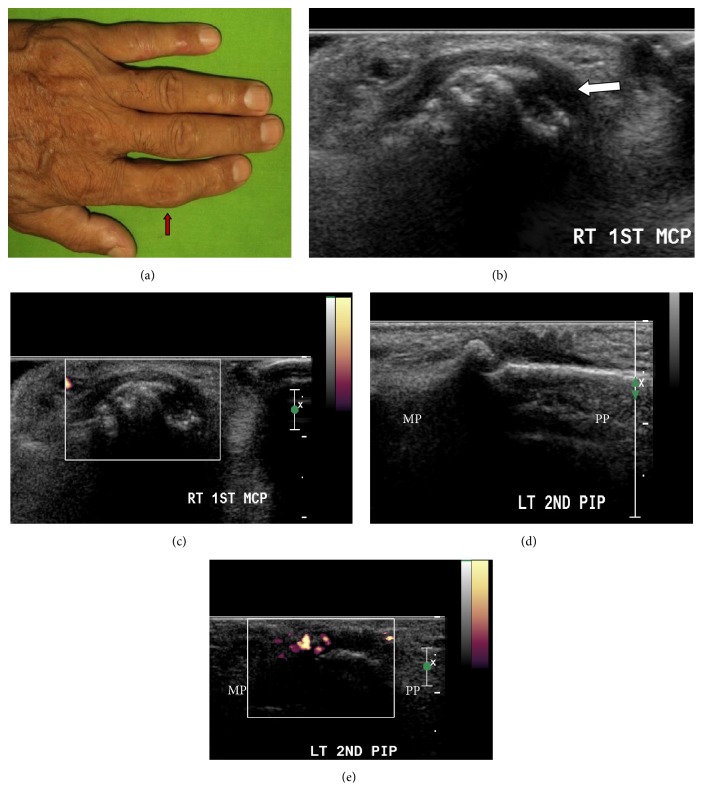
A 52-year-old male patient with PsA presented with pain in multiple joints of the hands in addition to other joints with swelling in left second PIP joint (a, arrow). DAS28 score was 4.78 (suggestive of moderate disease activity) at the time of presentation. US revealed extensive disease in multiple joints of the hands. The GS and PD scores were as follows: GSJC-12, GSJS-18, PDJC-4, and PDJS-8. Grade 2 GS synovial score with Grade 0 PD score in right first MCP joint (b and c). Grade 2 GS score with Grade 2 PD in left second PIP joint (d and e). Longitudinal view (d and e). Transverse view (b and c).

**Figure 4:**
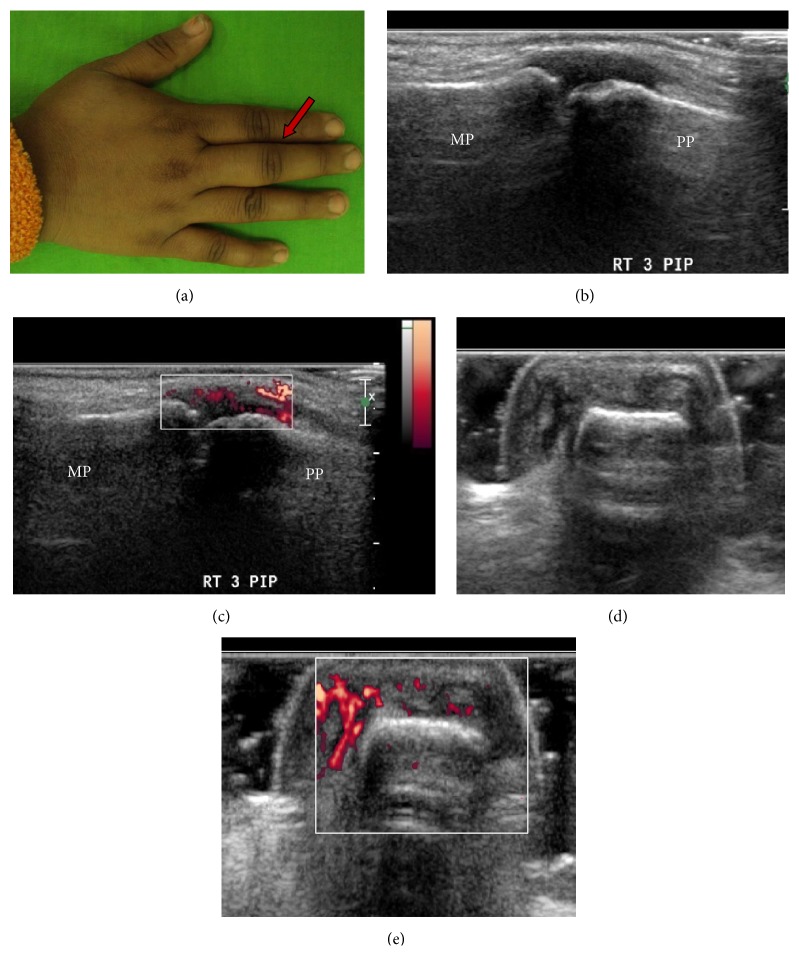
A 30-year-old female patient diagnosed as PsA presented with history of pain and swelling of right third PIP (arrow) (a). DAS28 score was 4.48 (suggestive of moderate disease activity). Ultrasound examination revealed Grade 2 synovial hypertrophy in right third PIP joint (b and d). PD evaluation showed increased signal (Grade 3) within the hypertrophied synovium in the joint (c and e). US scores derived were as follows: GSJC-11, GSJS-18, PDJC-3, and PDJS-7. Longitudinal view (b and c). Transverse view (d and e).

With GS, abnormal synovial thickening was observed in the joints of all the 30 patients (100%). Out of the scanned joints of the hand, it was seen in 182/840 joints (21.6%) and PD signal abnormality (suggested by scores or 1, 2, or 3) was seen in 25/840 joints and in 11/30 (36.7%) patients (Figures 3 and 4).

Out of 182 joints with abnormal synovial hypertrophy, 71 (39.01%) were MCP, 69 (37.91%) were PIP, and 42 (23.07%) were DIP joints. Thus, more synovial hypertrophy was detected by US than by clinical examination; however PD signal abnormality was noted in fewer number of joints than were clinically tender or swollen.

Soft tissue thickening was observed in 20/30 (66.7%) patients. None of the scanned joints revealed joint effusion. In only 2/30 (6.7%) patients, flexor tendonitis was observed. Periosteal reaction was observed in 10/30 (33.3%) patients. Erosions were observed in 9/30 (30%) patients, mostly in the DIP and PIP joints. There were no PD signs inside or near the erosions.

### 3.3. Correlation between Clinical and US Measures of Synovial Disease

**Figure 5:**
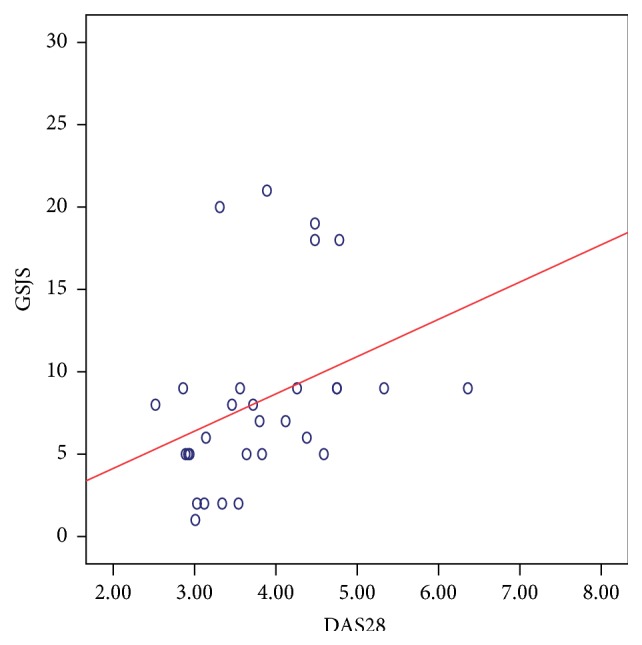
Relation between GSJS (taken from 28 MCP, PIP, and DIP joints) and DAS28 score in 30 patients with PsA. Spearman's *ρ*: 0.499; *P*: 0.005 suggesting a significant correlation.

**Figure 6:**
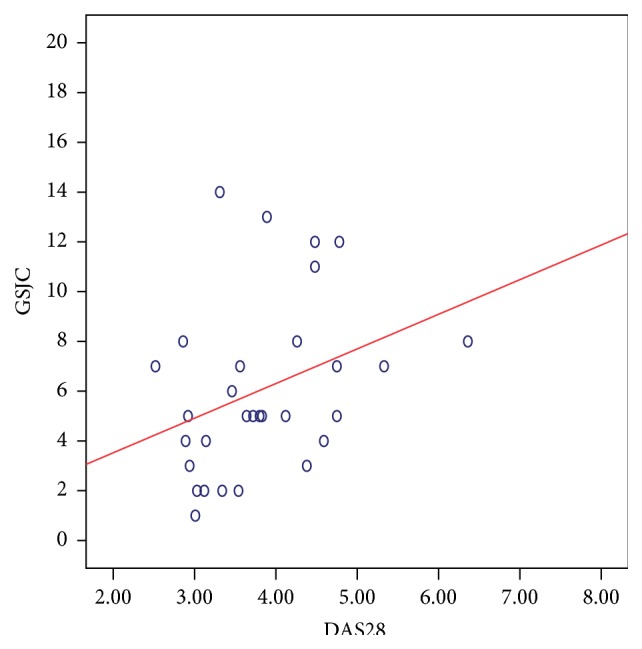
Relation between GSJC (taken from 28 MCP, PIP, and DIP joints) and DAS28 score in 30 patients with PsA. Spearman's *ρ*: 0.398; *P*: 0.029 suggesting a significant correlation.

**Figure 7:**
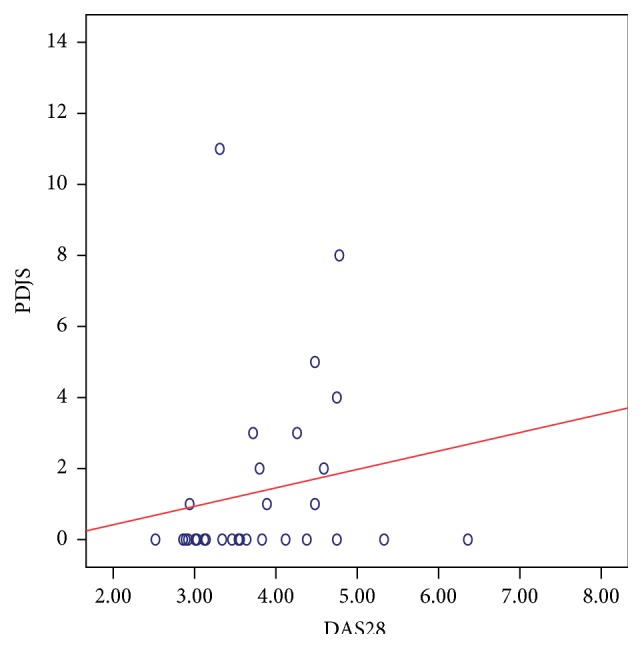
Relation between PDJS (taken from 28 MCP, PIP, and DIP joints) and DAS28 score in 30 patients with PsA. Spearman's *ρ*: 0.367; *P*: 0.046 suggesting a significant correlation.

There was a significant positive correlation between the DAS28 score and the composite US scores. The strongest relation was noted with the GSJS (Spearman's *ρ*: 0.499; *P*: 0.005) (Figure 5) and the GSJC (*ρ*: 0.398; *P*: 0.029) (Figure 6), and also significant relation was noted with the PDJS (*ρ*: 0.367; *P*: 0.046) (Figure 7) but only weaker relation was noted with the PDJC (*ρ*: 0.348; *P*: 0.060, NS).

**Figure 8:**
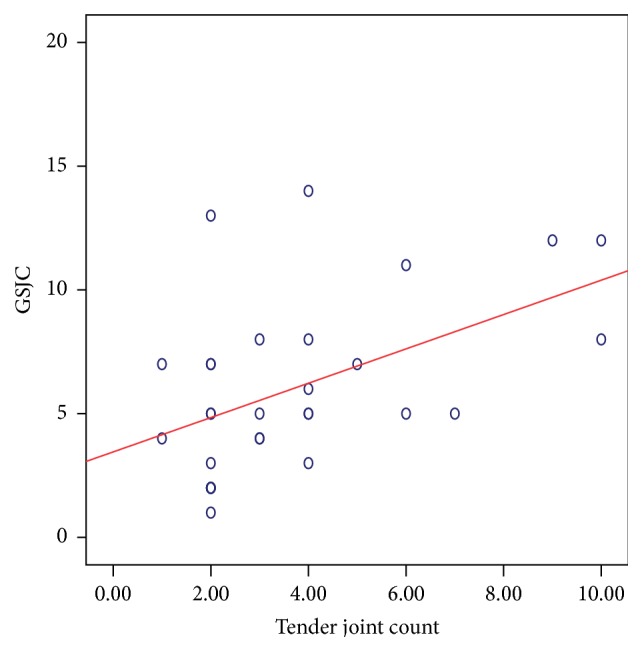
Relation between GSJC (taken from 28 MCP, PIP, and DIP joints) and TJC in 30 patients with PsA. Spearman's *ρ*: 0.474; *P*: 0.008 suggesting a significant correlation.

**Figure 9:**
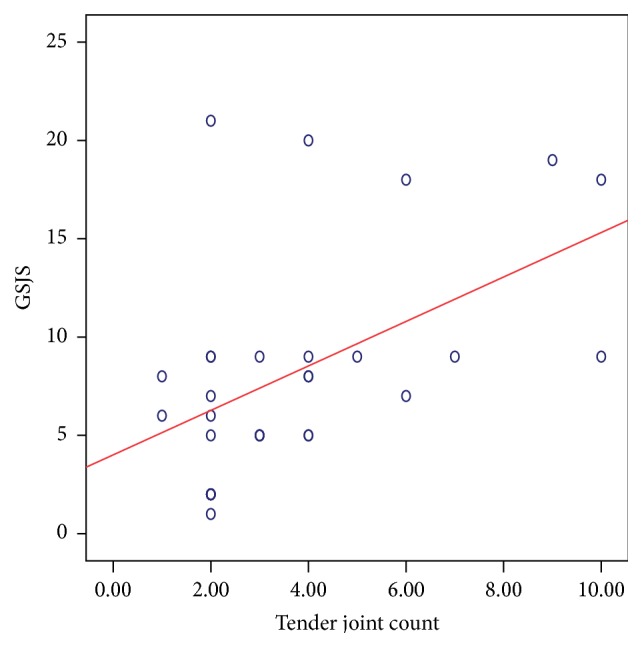
Relation between GSJS (taken from 28 MCP, PIP, and DIP joints) and TJC in 30 patients with PsA. Spearman's *ρ*: 0.484; *P*: 0.007 suggesting a significant correlation.

**Figure 10:**
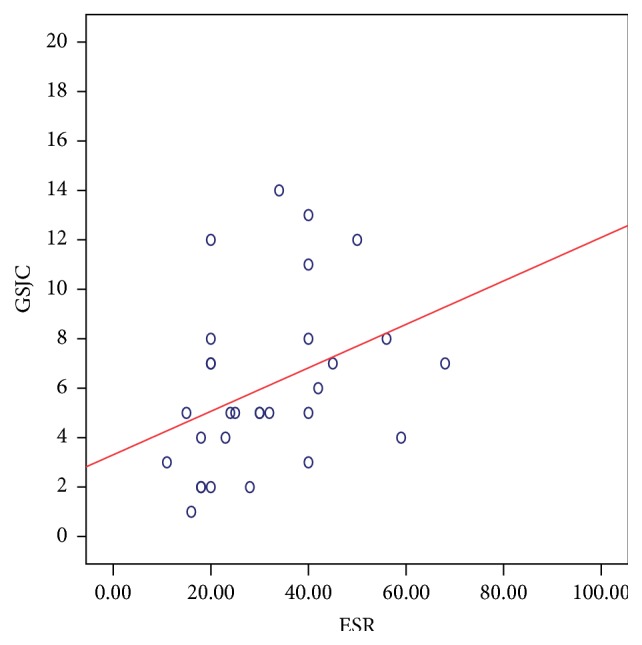
Relation between GSJC (taken from 28 MCP, PIP, and DIP joints) and ESR in 30 patients with PsA. Spearman's *ρ*: 0.478; *P*: 0.008 suggesting a significant correlation.

Correlation between the components of the DAS28 score [TJC, SJC, ESR, and VAS] and US measures was as follows: TJC is correlated positively with GSJC (*ρ*: 0.474; *P*: 0.008) (Figure 8), GSJS (*ρ*: 0.484; *P*: 0.007) (Figure 9), PDJC (*ρ*: 0.461; *P*: 0.010), and PDJS (*ρ*: 0.481; *P*: 0.007). There was no significant relation between the SJC and any of the GS or PD measures. ESR correlated with the GSJC (*ρ*: 0.478; *P*: 0.008) (Figure 10) and GSJS (*ρ*: 0.434; *P*: 0.017) and not with PDJC or PDJS. The VAS showed significant relation with GSJS (*ρ*: 0.541; *P*: 0.002), with GSJC (*ρ*: 0.423; *P*: 0.020), and with PDJC (*ρ*: 0.365; *P*: 0.047) and weaker relation with PDJS (*ρ*: 0.360; *P*: 0.051, NS).

On applying Mann-Whitney *U* test for comparison of US measures (GSJC, GSJS, PDJC, and PDJS), the difference between remission and low disease activity group and moderate disease activity group was statistically significant in terms of GSJC (*P*: 0.039), GSJS (*P*: 0.021), PDJC (*P*: 0.029), and PDJS (*P*: 0.027). Moderate disease activity zone patients demonstrated higher US measures as compared to the values within the remission and low disease activity zone patients.

However, no statistically significant difference was found between remission and low disease activity group and high disease activity group or between moderate disease activity group and high disease activity groups regarding the US measures. This was because of less number of patients (only 2) within the high disease activity zone, and hence the actual comparison between these groups could not be well evaluated.

## 4. Discussion

In our study we found various abnormalities on US evaluation of the joints which included abnormal synovial thickening, soft tissue thickening, tendonitis, periosteal reaction, erosions, and hypervascularity within the abnormal synovial thickening on Power Doppler. Out of four US measures, GSJC, GSJS, and PDJS demonstrated considerably significant correlation with DAS28 score.

MSUS with PD is routinely available, noninvasive, relatively inexpensive, patient friendly imaging method. It is a rapidly evolving technique in assessment of psoriatic arthritis. It assesses synovial tissue, joint effusions, and erosions. Power Doppler sonography depicts increased soft tissue vascularity and synovial inflammation. It allows for the characterization of early inflammatory changes in arthritis [13].

Several studies have recognized the efficiency of ultrasound in detecting inflammation in the joints of patients with PsA, in addition to the degree of structural damage [14].

US synovial hypertrophy was identified according to the OMERACT definitions and published descriptions of US pathology. Synovial hypertrophy is defined as abnormal hypoechoic (relative to subdermal fat but sometimes may be isoechoic or hyperechoic) intraarticular tissue that is nondisplaceable and poorly compressible and which may exhibit Doppler signal [15, 16].

Our study demonstrated that abnormal synovial thickening was observed in the joints of all the 30 patients (100%). Other studies have shown a variable and high percentage (up to 52% to 60%) of synovial abnormality in PsA [17–19].

It was seen in our study that synovial hypertrophy was more often detected by US than by the clinical examination which seems to be in line with previous studies [12, 20]. Another study has validated US in identifying the abnormalities involving the synovial tissue in the fingers and toes of patients with suspected PsA [21]. In a study by Wakefield et al., in 64% (51/80) of cases, US detected synovial inflammation in more number of joints than clinical examination [10].

Increased PD signal was seen in 25/840 joints corresponding to 11/30 (36.7%) patients. Out of 182 joints showing abnormal synovial hypertrophy, 25 (13.73%) joints showed PD signal abnormality.

Previous studies have revealed that the prevalence of PDUS synovitis was significantly higher in psoriatic patients than in controls [19].

A study by Caldarola et al. in 2011 found that most of the patients who had abnormalities on US also depicted vascular spots, suggesting active inflammation, in intra-articular and/or peritendinous spaces on PDUS, thereby providing additional information on disease activity. Twenty-nine of thirty-six patients who had grayscale US abnormalities suggestive of PsA also showed increased vascularity on PDUS in the abnormal synovial tissue [21].

The lower incidence of PD abnormality in the examined joints in our study can be attributed to the patient characteristics. Most of the patients were on treatment prior to assessment of the joints by PDUS which might have reduced the inflammation within the joints which generates the PD signal. Such an effect of treatment has been demonstrated in RA as well as PsA patients in a study by Backhaus et al. 2009, wherein the PD scores significantly decreased after 6 months of therapy [22].

A study by Weiner et al. 2008 has shown that US had a sensitivity of 40% and 57% for erosions when compared to radiography and MRI [23]. In addition studies by Backhaus et al. [22] and Weiner et al. [23] suggested that erosions are more frequently seen in PIP and DIP joints by US and radiography. This is in accordance with our data. PIP and DIP joints are also involved in primary OA and erosions might not be a specific finding. There were no PD signals inside or near the erosions. However, there was hypertrophied synovium identified in the region of erosion.

Prevalence of periosteal reaction in patients with PsA is up to 25%. In a previous study by Weiner et al., 10 out of 21 joints showing osteoproliferations on radiography also depicted them on US. On the contrary, US suggested osteoproliferative changes without corresponding changes in radiography in six joints. The reported sensitivity of US in comparison to the radiography in the detection of osteoproliferative changes was 10/21 (0.48) and the specificity was 163/169 (0.96) [23].

In our study, the presence of synovial thickening was scored on grayscale and Power Doppler into the Grades 0–3 as shown in Table 2. This semiquantitative scoring system introduced by Szkudlarek in 2003 has been widely used in other studies [24, 25]. Such a grading system has been used in previous studies by Backhaus et al. in which they have evaluated a novel 7-joint ultrasound score in daily rheumatologic practice. They did the study on 120 patients with RA (91%) and PsA (9%). They used the similar grading system for assessment of GS and PD findings [22].

Studies done in patients with RA have shown that GS and PD ultrasound measures have better reliability than the commonly used clinical indices in the evaluation of synovitis and that PD scores may be applied with greater accuracy than clinical scores of synovitis for treatment decisions [26]. The relationship between Power Doppler ultrasonography and clinical scores has not been extensively evaluated in psoriatic arthritis.

Using these scores, composite scores were derived as GSJC, GSJS, PDJC, and PDJS as has already been described. Such a composite scoring system has been used previously in evaluation of disease activity compared to clinical measures in patients with RA [11].

In our study, out of four US measures, GSJC, GSJS, and PDJS demonstrated considerably significant correlation with DAS28 score. Although GS measures showed stronger association than the PD measures, the PD score did correlate significantly and hence demonstrates its utility for the assessment of severity and hence an ongoing inflammation.

The GSJC, GSJS, PDJC, and PDJS were higher in the patients with moderate disease activity as compared to patients with remission and low disease activity. This implies that as the clinical severity score increases, the US scores also show increment.

In the US examination, we included the 28 joints of hands, rather than all those 28 joints that compose the DAS28 score, because PsA often involves DIP joints in addition to PIP and MCP joints. This may affect the analysis of the correlations between clinical data and US data in those cases that have synovitis involving the omitted joints that are knees, wrists, elbows, and shoulders. However, in the cohort studied, only those patients with complaints specific to hands were included and involvement of other joints which contribute to DAS28 was observed to be minimal.

The limitation of our study includes the use of two US machines, so different sensitivity of detecting changes by the US system would have affected the prevalence of various US findings in the hand joints. Lack of reference standard over an ultrasound examination is another limitation since ultrasound detects more abnormality than clinical exam but that may be due to ultrasound overdiagnosing abnormalities that are not clinically relevant.

## 5. Conclusion

Ultrasound is a useful modality for the objective assessment of PsA, which can detect joint inflammation to a larger extent than clinically expected. Ultrasound findings correlate well with clinical disease activity in patients with psoriatic arthritis. Hence, it may be said that ultrasound including PD can be used as a modality for assessment of severity of psoriatic arthritis in relation to the clinical scoring. Ultrasound including PD may provide useful information regarding the joint disease in the situations where the clinical assessments of severity as DAS28 or ESR are discordant.

## Figures and Tables

**Table 1:** Subjective GS and PD scoring system for US images of MCP, PIP, and DIP joints.

GS synovial score	PD score
0: absence of synovial hypertrophy	Absence of PD signal
1: small degree of synovial hypertrophy	Single vessel dots
2: moderate synovial hypertrophy	Confluent vessel dots over less than half the area of synovium
3: marked synovial hypertrophy	Confluent vessel dots over greater than half the area of synovium

**Table 2:** Demographic data of the patients.

Total number of patients	Male	Female	Mean age	Age range	Family history of psoriasis	Nail involvement	Number and percentage of patients on medical treatment			
30	16	14	38.97 ± 12.18 years	18–62 years	0	8 (26.7%)	None 5 (16.7%)	Methotrexate (MTX) 15 (50%)	MTX and Steroids 4 (13.3%)	NSAIDS 6 (20%)

**Table 3:** Range of clinical and ultrasound measures of synovial disease.

Disease activity measures	Minimum	Maximum	Median	Mean	SD (±)
TJC	1	10	3.00	3.77	2.487
SJC	0	9	1.00	1.67	2.249
ESR	11	68	29.00	31.40	14.407
VAS	8	80	20.00	26.67	17.980
DAS28	2.52	6.36	3.68	3.8267	0.86204
GSJC	1	14	5.00	6.07	3.483
GSJS	1	21	7.5	8.27	5.558
PDJC	0	7	0.00	0.83	1.533
PDJS	0	11	0.00	1.37	2.619
